# A Cross-Cultural Perspective on the Preference for Potential Effect: An Individual Participant Data (IPD) Meta-Analysis Approach

**DOI:** 10.1371/journal.pone.0124170

**Published:** 2015-03-25

**Authors:** Xiaomin Sun, Dan Xu, Fang Luo, Zihan Wei, Cong Wei, Gang Xue

**Affiliations:** 1 School of Psychology, Beijing Normal University, Beijing, 100875, China; 2 Department of Psychology, School of Education Science and Technology, Zhejiang University of Technology, Hangzhou, 310023, China; 3 Institute of Psychology, Chinese Academy of Sciences, Beijing, 100101, China; 4 Department of Public Administration, Chinese Academy of Governance, Beijing, 100089, China; Tilburg University, NETHERLANDS

## Abstract

A recent paper [Tormala ZL, Jia JS, Norton MI (2012). The preference for potential. Journal of personality and social psychology, 103: 567-583] demonstrated that persons often prefer potential rather than achievement when evaluating others, because information regarding potential evokes greater interest and processing, resulting in more favorable evaluations. This research aimed to expand on this finding by asking two questions: (a) Is the preference for potential effect replicable in other cultures? (b) Is there any other mechanism that accounts for this preference for potential? To answer these two questions, we replicated Tormala et al.’s study in multiple cities (17 studies with 1,128 participants) in China using an individual participant data (IPD) meta-analysis approach to test our hypothesis. Our results showed that the preference for potential effect found in the US is also robust in China. Moreover, we also found a pro-youth bias behind the preference for potential effect. To be specific, persons prefer a potential-oriented applicant rather than an achievement-oriented applicant, partially because they believe that the former is younger than the latter.

## Introduction

A dean of an academic institute is trying to find a suitable person for a tenure track position. Currently, there are two applicants: one has already published eight articles in high-impact journals in the past two years, and the other has the potential to do so in the coming two years. Which one, do you suppose, will the dean prefer to hire? Intuitively, the applicant with a record of remarkable achievement should be more impressive because potential is uncertain, while achievement is known. Usually persons responsible for hiring would prefer to evaluate an applicant based on clearly known factors rather than uncertain factors, given that all other conditions are equal. Surprisingly, Tormala, Jia, & Norton [1] demonstrated that this is not the case: persons often prefer potential rather than achievement when evaluating others. Uncertainty plays a key role in the preference for potential because uncertainty surrounding potential fosters greater interest and deeper processing, which in turn promotes more favorable evaluations [2].

This preference for potential effect is of great importance because it can shape attitudes and behaviors in a wide variety of domains, including organizational hiring, athletic recruiting, and undergraduate and graduate admissions. This study aimed to expand on the results of Tormala et al.’s study by asking two questions. First, is the preference for potential effect a culture-dependent phenomenon? Second, aside from the uncertainty causing deeper processing, is there any other mechanism contributing to the preference for potential effect? We will elaborate on these two questions below.

In Tormala et al.’s research in the United States, eight studies consistently showed that persons often prefer potential rather than achievement when evaluating others. Because culture often orients people toward particular manners of thinking, it plays an important role in shaping individual’s preferences. This led us to ask whether the preference for potential found in the United States could be generalized to other cultures. This is an open question that requires deep exploration, as the answers to it may be controversial. Take Chinese culture, for example.

First, considering the difference in social mobility in China compared to other countries, persons might prefer achievement instead of potential in China. China’s social mobility is low compared to the United States mainly because of the differences in the overall social statuses and occupational structures of these two countries. The Hukou registration system and locally funded education also account for the limited social mobility in China. Thus, it is relatively difficult in China for an individual to move upward in social status. In that regard, one’s potential would be regarded as less important because social structure greatly restricts a person’s development. However, the relatively high social mobility[3] in the United States encourages Americans to exert their potential and actualize their American dreams. One of the most important missions of the education system in the United States is to create conditions for each person to fulfill his/her potential. This suggests that culture of the United States is more likely to value potential. However, in China, a culture with low social mobility, it is possible that persons would be more likely to value achievement rather than potential.

Second, cultural differences in the tendency to avoid uncertainty between the United States and China might also cause the preference for potential effect to be non-existent or for an opposite preference for achievement to be present in China. Persons in Asia tend to avoid uncertainty more than persons in the United States [4,5]. Tormala et al.’s study showed that the uncertainty inherent in potential is crucial to the preference effect. Kupor et al. [2] showed that the preference for potential depends on individual and situational differences in tolerance for uncertainty and that this preference only emerges when the tolerance for uncertainty is high. Based on these results, the preference for potential might disappear or be altered among persons who find uncertainty aversive, such as those raised in the Chinese culture.

For these reasons, it is possible that the observed preference for potential effect found in the United States cannot be generalized to China. In other words, the Chinese may be more likely to prefer achievement rather than potential when evaluating others.

In contrast, previous cultural theories, such as the holistic-analytic thinking theory [6,7], have suggested that Chinese culture perceives change as cyclic, while culture of the United States perceives change as linear. In Chinese philosophy, Yin and Yang are concepts used to describe how apparently opposite or contrary forces are actually complementary, interconnected, and interdependent in the natural world. Consistent with this philosophy, the Chinese tend to believe that things will develop in the opposite direction when they reach extremes. Many Chinese sayings reflect this belief, such as, “the moon waxes only to wane,” and “water brims only to overflow.” Thus, it is plausible that the Chinese also have a preference for potential rather than achievement because they may believe that persons with a strong history of achievement might have fewer achievements in the future because they are reaching an extreme. In contrast, persons with potential are not approaching an extreme.

The cultural differences between the United States and China mentioned above led us to the reasonable question of whether the preference for potential effect found in the United States could be generalized to China.

If the social mobility and uncertainty avoidance accounts are true, we would expect our research results to support the following hypothesis:

H1a: The preference for potential effect is culture-dependent: persons raised in the Chinese culture prefer achievement instead of potential when evaluating others.

If the cyclic philosophy account is true, we would expect our research results to support the following hypothesis:

H1b: The preference for potential effect observed in the United States is generalizable to China: persons in China also prefer potential rather than achievement when evaluating others.

Next, we will address our second question: In addition to potential evoking greater interest and deeper processing, is there any other mechanism that account for the preference for potential effect?

Tormala et al.’s research showed that the preference for potential effect was not due to a pro-youth bias. They found no difference in the perceived age of high-potential and high-achievement applicant by participants. This finding is plausible because culture of the United States emphasizes that everyone has potential, regardless of his or her age. However, in Chinese culture, there is a popular lay belief that younger people have much more potential than older people. In *The Analects of Confucius* [8], there is a saying said that “a youth is to be regarded with respect, and the younger generation will surpass the older.” There are also many other proverbs that reflect the shared belief of the close connection between youth and potential. For example, “As in the Yangtze river, the waves behind drive on those before, so each new generation excels the old.” Therefore, we propose that the Chinese perceive the age of a person who has potential to be different from one who has a history of achievement: they believe that the former is younger than the latter. Thus, if there is a preference for potential effect in Chinese culture, this pro-youth bias is a mechanism that underlies it. Therefore, we propose the following hypotheses:

H2a: In China, potential-oriented applicants will be perceived to be younger than achievement-oriented applicants.

H2b: The preference of the Chinese for potential is affected by a pro-youth bias. To be specific, Chinese persons prefer a high-potential applicant to a high-achievement one, partially because they believe that the former is younger than the latter.

To examine these two hypotheses, we replicated Tormala et al.’s study using multiple samples in China and used an individual participant date (IPD) meta-analysis approach to analyze the data obtained. Instead of depending on summary statistics calculated for individual studies, the IPD meta-analysis approach utilizes all data from the included studies. According to Pigott [9], this approach may alleviate problems caused by missing data. With the original raw data, effect sizes can be computed using all available information, and analyses of effect size variation can use more detailed background characteristics of the study and the participants. Additionally, under many conditions, IPD meta-analyses have greater statistical power than aggregated data meta-analyses [9–11]. Because of these benefits of the IPD meta-analysis approach, we used it in this research.

The main purpose of this research was to clarify whether the preference for potential effect is culture-dependent and to explore alternative mechanisms behind it. The results will contribute to the development of theories in both decision-making and cultural psychology. Additionally, using the IPD meta-analysis approach to replicate Tormala and colleagues’ study is an innovative method to shed light on the research replicability issue in social psychology.

## Methods

### Samples

The Chinese Association of Social Psychology hosts a biannual Social Psychology Summer School in China. After reading Tormala et al.’s study, 18 researchers participating in the summer school were interested in it. We initiated a project to replicate this research. The project was reviewed and approved by the Academic Ethics Committee of the School of Psychology at Beijing Normal University (approval number: 2013028) before being conducted. The researchers independently replicated the third experiment of Tormala et al.’s study in ten cities in China. Each participant signed a written consent form.

One study was excluded because the study material it used was quite different from that of the other studies in some key aspects; for example, the gender of the applicants in the information participants received was female in the exclude study, while it was male for all other studies). The final 17 studies included in this meta-analysis were similar to the original experiment performed by Tormala et al. Only the background information of the applicants used in these 17 studies was allowed to be different from that of the original study. Of these 17 studies, nine directly used a translated version of Tormala et al.’s study materials (translated version), while the other 8 studies used altered materials that changed the applicants’ background information making it localized to Chinese context (localized version).

The sample size, gender ratio, mean age and location of data collection for each study’s sample are presented in Table 1. Individual participant data were obtained for all 17 studies. For participants’ age, 10 studies provided participant-level data, two studies only provided the mean age of the sample, and the other five studies failed to collect this information. For participants’ gender, 13 studies provided participant-level data, two studies provided the gender ratios of their samples, and the other two failed to collect this information. The number of participants in each study varied from 30 to 110.

**Table 1:** Study and sample characteristics.

Researcher	Sample size	Gender (% female)^a^	Age		Location	Background information
			*M* ^b^	*SD*		
Bai, BY	76	18.33%	22.36	0.87	Handan	localized
Chen, H	57	60.00%	20.24	1.44	Tianjin	translated
Geng, XW	79	NA	NA	NA	Yantai	translated
Guo, XL	58	60.00%^c^	21.00^d^	NA	Beijing	translated
Hao, J	71	90.14%	21.45	0.97	Guangzhou	localized
He, LN	64	76.56%	20.14	1.05	Guangzhou	translated
Ke, YN	69	72.46%	26.64	3.82	Beijing	localized
Lan, T	42	71.43%	26.17	4.30	Beijing	localized
Li, L	110	55.88%	NA	NA	Shanghai	translated
Li, WJ	87	74.71%	NA	NA	Yantai	localized
Luo, Y	48	NA	NA	NA	Beijing	localized
Tan, XY	30	50.00%	22.17	1.12	Qufu	translated
Wei, ZC	38	38.10%	NA	NA	Ningbo	localized
Xu, D	56	50.00%	21.95	0.84	Hangzhou	localized
Zhang, QM	78	50.00%	21.23	1.26	Yantai	translated
Zhang, QP	106	62.26%^c^	21.17^d^	0.97^e^	Guangzhou	translated
Zhou, J	59	62.07%	22.86	1.27	Wuhan	translated

^a^ The gender ratio was calculated after missing values in the study were replaced.

^b^ The mean age was calculated after missing values in the study were replaced.

^c^ Only gender ratio of the sample was provided without participant-level data.

^d^ Only the mean age of the sample was provided without participant-level data.

^e^ Only the SD of the sample was provided without participant-level data.

In total, 1,128 participants were recruited in these studies. They were from 10 different cities scattered throughout China, including cities located in southern China (e.g., Guangzhou); North China (e.g., Tianjin); coastal areas of China (e.g., Yantai); and inland areas of China (e.g., Handan). Some of the included cities were small-sized (e.g., Qufu) and some were large-sized (e.g., Beijing). Thus, these 17 studies included a rather representative sample of the Chinese culture.

### Manipulation

Replicating the third experiment of Tormala et al.’s study, each participant was asked to directly compare and evaluate two male applicants, A and B, for a managerial position with their information displayed on one paper side by side. The applicants’ background information, including their gender, date of birth, educational background, and internship experiences, were designed to be similar to each other.

The key difference between applicant A and B was their performance on two ostensible job tests, the Leadership Achievement Inventory (LAI) and the Assessment of Leadership Potential (ALP). The LAI was described as a measurement of an applicant’s currently demonstrated leadership performance, whereas the ALP was described as an estimate of an applicant’s future leadership performance. To manipulate which applicant was potential-oriented and which was achievement-oriented, we replicated the same test scores as Tormala et al.’s study. Applicant A was high (96/100) in potential while relatively moderate (83/100) in achievement, whereas applicant B was high (96/100) in achievement while relatively moderate in potential (83/100). Following Tormala et al.’s study, all studies were required to generated study materials with the background information of the potential-oriented and achievement-oriented applicants reversed to ensure that the background information varied systematically. Then, the two counterbalanced versions of the study materials were randomly distributed to the participants.

### Dependent Measures

After reading the information about the two applicants, participants were asked to complete a series of questions as dependent measurements. Following Tormala et al.’s study, we measured positive assessments of each applicant to test the preference for potential effect, measured negative assessments of each applicant to eliminate the extremity effect (the effect of evaluating high-potential individuals either more positively or more negatively), and measured the perceived age of each applicant to test for pro-youth bias. In addition, participants were asked to directly compare the two applicants based on their expected future performance by their 5th year at the company to test the preference for potential effect. They were also asked to compare currently which applicant’s resume is more objectively impressive to examine the study manipulation.

#### Positive assessments

The following 3 items were used to measure the positive assessments of the candidates: (1) If you were a manager at the company in question, how interested would you be in hiring Applicant A (Applicant B)? (2) How successful do you think Applicant A (Applicant B) will be in his career? and (3) Would hiring Applicant A (Applicant B) at the company be a good decision or a bad one? All three items contained scales ranging from 1 to 9, with higher values indicating more favorable assessments. These items and evaluation scales were exactly the same as those used in Tormala et al.’s study. Then, we averaged the results of the positive assessments to form composite indices for each as a total positive assessment of the candidates.

Given that each primary study used a within-subject design, we computed the δ-pos value as the composite positive assessment score for applicant A minus the composite positive assessment score for applicant B. According to the job testing score, applicant A is potential-oriented and applicant B is achievement-oriented. Thus, a significantly larger than zero δ-pos value indicated that the potential-oriented applicant was evaluated more favorably than the achievement-oriented applicant.

#### Negative assessments

Following the positive assessments, we included negative assessments of the two applicants. Participants were asked to indicate the following: (1) the likelihood that each applicant would turn out to be a failure and (2) the likelihood that each applicant would be a disappointment in the long run. The scales for these assessments ranged from 1 (not likely at all, very low) to 9 (very likely, very high). These items and scales were exactly the same as those used in Tormala et al.’s research. Then, we averaged the two items to form composite indices for the negative assessments of each applicant.

Similar to the δ-pos values, we computed the δ-neg value as the composite negative assessment score for applicant A minus the composite negative assessment score for applicant B. Thus, a significantly smaller than zero δ-neg value indicated that the potential-oriented applicant was evaluated less negatively than the achievement-oriented applicant.

#### Age perception

The participants’ age perceptions of the two applicants were measured by asking the participants to indicate how young or old they believed each applicant was on a scale ranging from 1 (very young) to 9 (very old). To compute the δ-age-per value, we first recoded the scores for applicant A and B (i.e., 1 was recoded as 9, 2 was recoded as 8, and so forth), and, then, used the recoded scores for applicant A minus the recoded scores for B. Thus, a significantly larger than zero δ-age-per value indicated that the persons believed that the potential-oriented applicant was younger.

#### Five-year performance

Following Tormala et al.’s study, one item was used to directly compare applicant A and applicant B on their performance by their 5th year at the company, with a scale that also ranged from 1 (applicant A would definitely perform better) to 9 (applicant B would definitely perform better). To make the index more understandable, we first recoded the raw scores (i.e., 1 was recoded as 9, 2 was recoded as 8, and so forth). Then, we computed the δ-five-year value using the recoded raw score minus 5. A significantly larger than zero δ-five-year value indicated that the persons believed that high-potential applicant would perform better in the next five years than the high-achievement applicant.

#### Resume impressiveness

Finally, as a manipulation check, participants were asked to evaluate which applicant had a more objectively impressive resume at present, with a scale ranging from 1 (definitely applicant A) to 9 (definitely applicant B). We computed the δ-CV value using the raw score minus 5. A significantly larger than zero δ-CV value indicated that the participant believed the achievement-oriented applicant was more successful than the potential-orientated applicant at the current moment in time.

### Data analysis

Seventeen studies were eligible for inclusion in the meta-analysis because they all examined the preference for potential effect by the same manipulation, the same measurements and the same scales. As mentioned previously, the only difference among these 17 studies was the study materials used to characterize the background information of the applicants (translated version versus localized version).

Due to the diversification of studies in regards to participant characteristics and study materials, one stage random effect meta-analyses were applied to the IPD collected by these seventeen studies with restricted maximum likelihood estimation [9–15]. One-stage models have advantages over two-stage models when investigating participant-level sources of heterogeneity, as both participant-level characteristics and study-level variables can be incorporated into the model [16]. As a within-subject design, the preference for potential effect and the meditational effect of pro-youth bias were examined, following the methodology described by Judd and Kenny[17,18]. At the participant-level, the participant characteristics (i.e., age and gender) and the counterbalancing condition were treated as stable concomitant variables, while age perception was treated as a varying concomitant variable. The background information (translated version vs. localized version) of each study was treated as a study-level variable.

Three multilevel models were employed in the data analysis. The first model investigated the preference for potential effect (δ-pos, δ-neg and δ-five-year) and the pro-youth effect (δ-age-per) after adjustment for the participants’ characteristics (age and gender) and the counterbalancing condition. The second model tested the mediation effect of age perception on the preference for potential effect. Finally, to eliminate the possibility that different types of background information might have affected the preference for potential effect, when there was a significant difference between studies, background information was entered into the original model as a study-level variable. Before these steps of multilevel analysis were performed, the validity of our manipulation and the reliability of each study were examined.

Missing values in each study were replaced by the group mean of the study variables (the ratios of missing values were under 5% in all studies). For variable missing in one study, if the study mean was available, all individual values were assigned the value of the study mean. If the study mean was inaccessible, all individual values were assigned the grand mean of all studies.

All statistical analyses were performed using SPSS 22.0 and HLM 7.

## Results

### Reliability and validity check

For the positive assessments, three items were highly correlated with each other for both applicant A (*M*_*d*_ *α* = 0.83) and applicant B (*M*_*d*_ *α* = 0.79). For the negative assessments, two items were moderately correlated for both applicant A (*M*_*d*_ *α* = 0.71) and applicant B (*M*_*d*_ *α* = 0.71). The reliabilities of each study are listed in Table 2.

**Table 2:** Reliability and descriptive statistics of 17 studies on dependent measures.

Researcher	Positive assessments						Negative assessments						Five-year performance	
	Applicant A (potential-oriented)			Applicant B (achievement-oriented)			Applicant A (potential-oriented)			Applicant B (achievement-oriented)				
	*α*	*M*	*SD*	*α*	*M*	*SD*	*A*	*M*	*SD*	*α*	*M*	*SD*	*M*	*SD*
Bai, BY	0.87	6.43	1.67	0.79	6.20	1.38	0.73	4.09	1.49	0.66	4.71	1.50	4.07	2.73
Chen, H	0.86	7.19	1.21	0.81	6.92	1.00	0.55	3.57	1.33	0.71	3.97	1.41	4.58	1.93
Geng, XW	0.79	6.76	1.17	0.84	6.50	1.21	0.72	3.70	1.34	0.68	4.18	1.45	4.00	2.20
Guo, XL	0.77	7.30	1.23	0.65	6.91	1.12	0.58	3.10	1.43	0.39	3.81	1.23	4.28	1.86
Hao, J	0.86	7.11	1.39	0.87	7.00	1.36	0.55	3.77	1.37	0.64	4.04	1.38	5.73	1.99
He, LN	0.77	6.86	1.02	0.79	6.66	1.00	0.76	3.90	1.26	0.86	4.01	1.23	4.73	1.36
Ke, YN	0.88	6.58	1.44	0.86	6.29	1.35	0.54	3.81	1.34	0.74	4.02	1.40	4.41	1.90
Lan, T	0.75	6.16	0.99	0.72	5.72	1.12	0.74	3.62	1.20	0.77	3.86	1.17	4.36	1.78
Li, L	0.85	6.84	1.30	0.79	6.54	1.19	0.81	4.41	1.56	0.79	4.56	1.55	4.44	1.91
Li, WJ	0.42^a^	6.68^a^	1.67^a^	0.72	6.33	1.41	0.53	3.56	1.48	0.65	4.45	1.70	3.98	2.65
Luo, Y	0.72	6.97	1.11	0.83	6.44	0.99	0.77	3.63	1.25	0.79	4.12	0.49	4.50	2.00
Tan, XY	0.86	6.64	1.60	0.80	6.00	1.62	0.84	4.36	1.86	0.84	4.58	1.59	4.38	2.06
Wei, ZC	0.90	7.04	1.22	0.82	6.75	0.99	0.49	3.39	1.19	0.67	3.74	1.28	4.18	2.17
Xu, D	0.83	7.24	1.09	0.84	6.87	1.17	0.81	3.43	1.46	0.73	3.51	1.34	3.86	1.85
Zhang, QM	0.81	6.74	1.39	0.65	6.81	1.13	0.71	4.40	1.78	0.77	4.30	1.69	4.62	2.55
Zhang, QP	0.86	6.73	1.30	0.77	6.41	1.01	0.55	4.10	1.38	0.50	4.77	1.33	4.07	2.14
Zhou, J	0.79	6.51	1.16	0.79	6.53	1.11	0.55	3.56	1.08	0.58	4.09	1.38	5.15	2.24

*Note*: Raw means for Five-year performance are presented; values below 5 indicate a relative preference for potential over achievement.

^a^The Cronbach's alpha, mean and SD of this measurement is calculated based on two items instead of three because participant’s responses on the first item of the positive assessment measures were lost in this study.

IPD random effect meta-analyses were used to test the validity of our manipulation using multilevel models. The δ-CV was set as the outcome variable adjusted for participant age and gender and the counterbalancing condition. One study was excluded from the dataset for this analysis because it didn’t collect data on CV impressiveness. The results showed that there was no significant difference in the participants’ impressions towards the resumes of applicants A and B’s resume (0.109 (95%CI -0.198; 0.417)). This suggests that our manipulation was valid and that the information provided in the resumes of applicants A and B was successfully designed to be roughly equal.

### Fixed and random effect of the preference for potential

IPD random effect meta-analyses were used to test the preference for potential effect and age perception bias using multilevel models. The δ-pos, δ-neg, δ-five-year and δ-age-per values were set as outcome variables, and the fixed and random effects of intercept for each variable were examined, controlling for the participant gender and age and the counterbalancing condition as group-centered, participant-level variables, assuming a fixed effect.

As shown in Table 3, the potential-oriented applicant was more favorable to people than the achievement-oriented applicant based on positive assessments (0.270 (%95CI 0.178; 0.361)), and people generally believed that the high potential applicant was more likely to perform better than the high-achievement applicant by his fifth year in the company (0.701 (%95CI 0.571; 0.830)). These results are consistent with Tormala et al.’s findings. However, our results also showed that people evaluated the potential-oriented applicant less negatively than the achievement-oriented applicant (-0.377 (%95CI -0.511; -0.244)). This finding is different from that of Tormala et al., who found no difference in the negative assessments of the two applicants. Our results suggest that the Chinese prefer potential both from a positive and negative viewpoint. The above findings generally support H1b.

**Table 3:** The preference for potential effect adjusted for participant gender and age and counterbalancing condition.

Outcome	n participants (n studies)	The preference for potential^a^ ES (95% CI)	Gender^b^ ES (95% CI)	Age^b^ ES (95% CI)	Counterbalance^b^ ES (95% CI)	ICC	U_0_ ^c^
δ-pos	1128(17)	0.270(0.178, 0.361)***	0.098(-0.143, 0.340)	-0.035(-0.098, 0.029)	0.036(-0.192, 0.264)	0.000	0.000
δ-neg	1128(17)	-0.377(-0.511, -0.244)***	-0.023(-0.262, 0.216)	-0.006(-0.069, 0.057)	0.028(-0.198, 0.254)	0.016	0.040**
δ-five-year	1128(17)	0.701(0.571, 0.830)***	0.174(-0.151, 0.500)	-0.005(-0.091, 0.080)	0.267(-0.040, 0.575)	0.001	0.006
δ-age-per	1080(16^d^)	0.324(0.216, 0.432)***	-0.017(-0.232, 0.197)	-0.053(-0.110, 0.003)	0.085(-0.117, 0.288)	0.011	0.020

*Note*: *CI* Confidence Interval, *ES* Effect size, *N* Number, *ICC* Intraclass correlation coefficient.

*** p<.001(2-tailed).

** p<.01(2-tailed).

* p<.05(2-tailed).

^a^ ES of the preference for potential after controlling for the participant’s gender and age and counterbalancing condition, which is the fixed effect of intercept.

^b^ ES of the predictors, i.e., gender and age and counterbalancing condition, which is the fixed effect.

^c^ Between-group variance.

^d^ One study was excluded from this part of the data analysis because it failed to collect participants’ age perception.

Moreover, although Tormala et al.’s research did not find a significant difference between the perceived age of the high-potential applicant and that of the high-achievement applicant, our meta-analysis revealed that the Chinese believe high-potential applicant is younger than high-achievement applicant (0.324 (%95CI 0.216,0.432)), even though their actual ages are similar according to the information in their resume. This result supports H2a.

For the δ-pos assessments δ-five-year and δ-age-per, the random effects were not significant, which indicate homogeneity in these studies for these variables. However, for the δ-neg assessments, the random effects were significant (U_0_ = 0.04, p<.01), which indicate heterogeneity between the studies for these measures.

For counterbalancing condition, 14 studies ran counterbalanced versions in their experiments, among which 11 provided participant-level data; and the other three failed to run counterbalanced versions. As showed in Table 3, the fixed effects of participant gender, age, and counterbalancing condition are not significant for all assessments, including positive assessments, negative assessments, five-years performance and perceived age, which indicate that participant characteristics and counterbalancing condition do not affect the preference for potential effect.

### Mediation effect of age perception bias on the preference for potential

#### Mediation analysis of age perception bias

Because the Chinese believe that the potential-oriented applicant was younger, could the observed differences in the positive assessments, negative assessments and five-years performance assessments between the potential-oriented and achievement-oriented candidates be due to this age perception bias? The δ-age-per value as an uncentered participant-level variable, assuming a fixed effect, was included in multilevel models to predict the outcome variables (δ-pos, δ-neg and δ-five-year).

As seen in Table 4, our results showed that δ-age-per significantly predicted the outcome variables, with the effects of 0.099 (95%CI 0.034; 0.164), -0.066 (95%CI -0.131; -0.001), and 0.210 (95%CI 0.123; 0.298) for the δ-pos, δ-neg and δ-five-year variables, respectively. Although the effects of preference for potential are smaller than basic models due to adjustment of group mean of δ-age-per, they are still significant with 0.237 (95%CI 0.143; 0.331), -0.356 (95%CI -0.490; -0.222) and 0.632 (95%CI 0.500; 0.763) for the δ-pos, δ-neg and δ-five-year variables, respectively. According to Judd and Kenny (2001), these results suggest that the preference for potential effect observed in this study could be partially explained by a pro-youth bias. In other words, Chinese persons prefer potential-oriented candidate, partially because they believe that potential-oriented candidate is younger. These findings support H2b.

**Table 4:** The effect of pro-youth bias on preference for potential.

Outcome	n participants (n studies)	Preference for potential^a^ ES (95% CI)	δ-age-per^a^ ES (95% CI)	Gender ES (95% CI)	Age ES (95% CI)	Counterbalance ES (95% CI)	ICC	U_0_
δ-pos	1128(17)	0.237(0.143, 0.331)***	0.099(0.034, 0.164)**	0.100(-0.140, 0.340)	-0.029(-0.093, 0.034)	0.028(-0.200, 0.255)	0.000	0.000
δ-neg	1128(17)	-0.356(-0.490, -0.222)***	-0.066(-0.131, -0.001)*	-0.024(-0.263, 0.215)	-0.01(-0.073, 0.053)	0.033(-0.192, 0.259)	0.016	0.039**
δ-five-year	1128(17)	0.632(0.500, 0.763)***	0.210(0.123, 0.298)***	0.178(-0.145, 0.500)	0.006(-0.079, 0.091)	0.25(-0.055, 0.554)	0.001	0.006

*** p<001(2-tailed).

** p<01(2-tailed).

* p<05(2-tailed).

^a^ ES of age-perception bias as the predictor, which is the fixed effect.

Other indices are the same as Table 3.

#### Homogeneity of the preference for potential between studies

Table 3 shows that there were no significant difference between the studies on δ-pos, δ-five-year and δ-age-per. However, we did find a significant difference between the studies for δ-neg (U_0_ = 0.040, p<. 01). Could this study-level difference be explained by the difference in background information between studies? The translated version was coded as 0, and the localized version was coded as 1. The background information variable was added to the multilevel models as a study-level variable to predict the intercept of the outcome variables. Table 5 shows that the effect of using the translated version versus the localized version as background information was not significant (-0.061 (95% CI -0.335; 0.212), indicating that the different types of background information did not affect the preference for potential effect.

**Table 5:** The effect of background information as a study-level variable on preference for potential.

Outcome	n participants (n studies)	The preference for potential^a^ ES (95% CI)	Gender ES (95% CI)	Age ES (95% CI)	Counterbalance ES (95% CI)	Background Information ES (95% CI)
δ-neg	1128(17)	-0.349(-0.534, -0.164)**	-0.023(-0.262, 0.216)	-0.006(-0.069, 0.057)	0.028(-0.198, 0.254)	-0.061(-0.335, 0.212)

** p<01(2-tailed).

^a^ ES of the preference for potential after controlling participant characteristics, counterbalancing condition, age perception bias as participant-level variables and background information as study-level variable.

Other indices are the same as Table 3.

Furthermore, although the between-group variance in δ-neg was significant, the ICC was very small (ICC = 0.016), indicating that these 17 studies were quite homogeneous (see Table 3).

## Discussion

The main goal of this study was to extend Tormala et al’s research by asking two questions: Is the effect of preference for potential effect replicable in other cultures? Is there any other mechanism that accounts for the preference for potential effect?

In answer to the first question, IPD meta-analysis results showed that the preference for potential effect is replicable in the Chinese culture. In both Tormala et al.’s study and the 17 studies in this research, the potential-orientated applicant received higher positive assessments and was expected to outperform the achievement-oriented applicant by his fifth year at the company.

However, the preference for potential is culture-dependent to some degree. In Tormala et al.’s study, there were no differences between the negative assessments of the two applicants. However, we found that Chinese people also preferred the high-potential applicant from the negative viewpoint; they viewed the applicant with high-potential to be less likely to fail and less likely to be a disappointment than the high-achievement applicant.

In answer to the second question, the IPD meta-analysis results suggested that there are cultural differences in the mechanisms behind the preference for potential effect. Our results indicated that the pro-youth bias could be one of multiple mechanisms that underlie the potential preference in the Chinese culture, while Tormala et al.’s study suggested that this is not the case in the United States. In contrast to Americans, the Chinese prefer potential-oriented applicant rather than achievement-oriented applicant, partially because they believe that the former is younger than the latter. This result is quite interesting and should remind cultural psychology researchers that particular phenomenon found to exist across different cultures may have different causes between the different cultures.

The main contribution of our research is that we expanded on the findings of Tormala et al.’s study from the perspective of cultural psychology. We found that the preference for potential effect is robust in China, while the mechanisms behind it might be different. There are also some important practical implications of our findings. When applying for a job in China, it is better for an applicant to highlight his/her potential instead of his/her previous achievements. In doing so, the applicant is perceived as significantly younger, and normally Chinese persons prefer younger applicant to older ones, given that all other aspects are equal. The findings of this research are not only important in the context of personnel selection, but also relevant to business and social contexts, such as international negotiation and marketing, when different strategies of persuasion could result in greatly different outcomes.

This study also provides a possible approach to handle the replicability issues in psychology research. In recent years, an increasing amount of researchers have emphasized the importance of result replicability in psychology research [19–21]. Without doubt, replicability is one of the most important tenets of science. The successful replication of results generates greater confidence in the veracity of a predicted effect while the failure to replicate results directs us to further explore the psychological mechanisms that underlie the effect or its boundary conditions [21]. However, how to make psychological research findings more robust is still under debate. Our research was an attempt to further this aim. By replicating Tormala et al.’s original research in 17 different samples and conducting an IPD meta-analysis, we showed that the effect of potential preference is robust in the Chinese culture.

## Limitations and Future Research Directions

According to Tormala et al. [1] and Kupor et al. [2], the generation of uncertainty by potential, which causes great interest and deeper processing, is the main reason for the preference for potential effect. This research found that pro-youth bias is another mechanism behind the preference for potential effect. In addition to these two mechanisms, do any other factors account for this phenomenon? This is an intriguing question and further research is warranted.

In this research, we found some cultural differences in the preference for potential effect. According to the dynamic constructive approach, culture is a network of domain-specific cognitive structures, including theories, beliefs and assumptions, and cultural differences in cognition, affect, and behaviors are mediated by some domain-specific lay theories, beliefs and assumptions [22,23]. In this study, we did not examine how culture affects the preference for potential. Why did the Chinese prefer people with potential from a negative viewpoint, while Americans did not? These are all interesting issues that could be explored in future studies.

There are some limitations in this research that future studies could pay close attention to. First, the external validity of our results could have been improved if we used actual managers instead of students as our research participants. Future studies could address this issue using different scenarios in various settings with diverse samples. Secondly, the reliabilities of the negative assessments for some of the included studies were not sufficient. A possible reason for this is that the Chinese views “failure” and “disappointment” differently. “Failure” is an objective outcome, while “disappointment” carries with a connotation of internal attribution. Although both are negative assessments, they might be affected by differences in persons’ processes of judgment and evaluation. Future research could examine this further. Thirdly, in this research, we replicated Tormala et al.’s study in 17 different samples, and used IPD meta-analysis to analyze the data. If there were more studies, the random effect of age-perception bias, gender and age of participants could have also been examined. Finally, this study demonstrates that the preference for potential effect found in the United States is also robust in China. More evidences are needed when generalizing the effects to other cultures.
